# Marine-Derived Compounds for the Potential Treatment of Glucocorticoid Resistance in Severe Asthma

**DOI:** 10.3390/md19110586

**Published:** 2021-10-20

**Authors:** Cristina Mihaela Ghiciuc, Andrei Gheorghe Vicovan, Celina Silvia Stafie, Sabina Antonela Antoniu, Paraschiva Postolache

**Affiliations:** 1 Department of Morpho-Functional Sciences II—Pharmacology and Clinical Pharmacology, Faculty of Medicine, Grigore T. Popa University of Medicine and Pharmacy of Iași, 16 Universitatii Street, 700115 Iasi, Romania; 2 Department of Preventive Medicine and Interdisciplinarity—Family Medicine Discipline, Faculty of Medicine, Grigore T. Popa University of Medicine and Pharmacy of Iasi, 16 Universitatii Street, 700115 Iasi, Romania; celina.stafie@umfiasi.ro; 3 Department of Medicine II—Palliative Care Nursing, Grigore T. Popa University of Medicine and Pharmacy of Iasi, 16 Universitatii Street, 700115 Iasi, Romania; sabina.antoniu@umfiasi.ro; 4 Department of Medicine I—Pulmonary Rehabilitation Clinic, Grigore T. Popa University of Medicine and Pharmacy of Iasi, 16 Universitatii Street, 700115 Iasi, Romania; paraschiva.postolache@umfiasi.ro

**Keywords:** marine-derived compounds, glucocorticoid-resistant, severe asthma, drug development, biomedical applications

## Abstract

One of the challenges to the management of severe asthma is the poor therapeutic response to treatment with glucocorticosteroids. Compounds derived from marine sources have received increasing interest in recent years due to their prominent biologically active properties for biomedical applications, as well as their sustainability and safety for drug development. Based on the pathobiological features associated with glucocorticoid resistance in severe asthma, many studies have already described many glucocorticoid resistance mechanisms as potential therapeutic targets. On the other hand, in the last decade, many studies described the potentially anti-inflammatory effects of marine-derived biologically active compounds. Analyzing the underlying anti-inflammatory mechanisms of action for these marine-derived biologically active compounds, we observed some of the targeted pathogenic molecular mechanisms similar to those described in glucocorticoid (GC) resistant asthma. This article gathers the marine-derived compounds targeting pathogenic molecular mechanism involved in GC resistant asthma and provides a basis for the development of effective marine-derived drugs.

## 1. Introduction

Asthma is a chronic inflammatory disease of the lower airways characterized by airway hyperresponsiveness and remodeling, leading to wheeze, cough, chest tightness, and difficulty in breathing. The prevalence of asthma is still increasing, while the potential risk factors for asthma seems to make equal contributions [1]. Although among the population of adults with asthma only 3% to 10% are classified as suffering from severe asthma [2,3], the costs of healthcare per patient are higher than those for stroke, type 2 diabetes, or chronic obstructive pulmonary disease (COPD) [4]. According to the current guidelines [5], difficult-to-control asthma is asthma that is uncontrolled despite treatment with high-dose inhaled glucocorticoids (ICS) combined with long-acting β_2_-agonists or other controllers, or that requires such treatment to maintain good symptom control and reduce exacerbation; severe asthma is considered a subset of difficult-to-control asthma that is uncontrolled despite adherence to maximal optimized Step 4 or Step 5 therapy and treatment of contributory factors, or that worsens when high-dose treatment is reduced.

Glucocorticoid resistance in the main challenge of severe asthma. A common feature of patients with severe asthma is their poor response to high doses of ICS and then systemic glucocorticoids (GCs). This inadequate response is called steroid-unresponsive or GC resistance. These patients, however, may respond well to β2-adrenergic agonist-mediated vasodilation; but if after 2 weeks of appropriate-dose steroid treatment the improvements in forced expiratory volume in 1 s (FEV1) are <15%, then the asthma is defined as GC resistant [6,7,8].

Compounds derived from marine sources have received increasing interest in recent years due to their prominent biologically active properties for biomedical applications, and to their being a new and safe source for drug development [9,10,11,12,13]. New compounds were recently reviewed and proposed as potential treatments for different diseases, such as cancer [14], cardiovascular diseases [15], rheumatoid arthritis [16], neurological diseases [17], and others.

Many marine-derived biologically active compounds target pathogenic molecular mechanisms common to those described in GC-resistant asthma; therefore, we sought in this review to gather the marine-derived compounds targeting the pathogenic molecular mechanism involved in GC resistant asthma and to provide a basis for the development of effective marine-derived drugs.

## 2. Pathobiological Features Associated with Glucocorticoid Resistance

In practice, the diagnosis of GC resistance in asthma is based on the clinical history and evaluation of respiratory function after sufficient steroid treatments. Often, patients receive increasing doses of steroids for extended periods, until it is recognized that this is ineffective for treating their severity of asthma. The toxic side effects of long-term high-dose steroids are well known for increasing susceptibility to infections, cardiovascular disease, hyperglycemia, and osteoporosis. Although there are currently no clinically accepted biomarkers or phenotypes for resistance, some studies identified asthma phenotypes associated with GC resistance [5,18,19], as depicted in Table 1.

## 3. Mechanisms of Glucocorticoid Resistance as Potential Therapeutic Targets 

### 3.1. Mechanisms of Action of Glucocorticoids

Although the topic has been extensively reviewed by Keenan et al. [20] and many others [7,21,22,23], before delving into the altered cellular and molecular basis of signaling that leads to GCs resistance, it is important to review the heterogenous mechanisms of action by which GCs exert their downstream effect. 

GCs have been extensively used in many diseases for a long time, but their molecular mechanisms of action are still not completely understood. GCs bind on the intracellular glucocorticoid receptors (GRs) of the target cell. There are two major variants of GRs with different C-terminal domains: GR-α, and GR-β. GR-α isoform-bind to GCs and affect GR signaling pathways through various post-translational modifications, such as phosphorylation, acetylation, and other modifications [24], while GR-β is unable to bind to GCs and cannot affect GC-induced modification. GR-β probably regulates GC activity, antagonizes GR-α isoform, and regulates GR-α/β heterodimers [25,26].

Genomic mechanisms are mediated by binding to GRs in the cytoplasm and further translocation of the GC/GR complex into the nucleus, while non-genomic mechanisms are mediated through specific interaction with GRs, or nonspecific interactions with the cell membrane [27]. Intracytoplasmic GRs present in inactive forms, in a protein complex, and attached to a chaperone protein. The dissociation of GR and the dissociation of chaperone protein upon activation allow the translocation of GR into the nucleus [28]. Inside the nucleus, the GC/GR complex regulates up to 20% of genes expressed by immune cells by trans-repressing inflammatory genes and stimulating the transcription of anti-inflammatory genes, leading to the reduced activation, recruitment, and survival of inflammatory and epithelial cells [29,30,31]; it also regulates mRNA stability [32] and the immunomodulatory function of smooth muscle cells and airway remodeling in asthma [33].

High concentrations of GCs exert non-genomic actions; inhibit the degranulation of mast cells through the stabilization of the plasma membrane or through a reduction in [Ca2+] elevation [34]; and promote anti-inflammatory effects through negative interference with MAPK signaling pathways [35].

### 3.2. Glucocorticoids Resistance: Cellular and Molecular Basis

Decreased GC responsiveness can be inherited or acquired. In the case of inherited decreased GC responsiveness, GC insensitivity most probably is not caused by a singular genetic mutation and involves a range of genetic variations. Some of the involved genes have already been determined [36,37,38] and are not the aim of our study.

The research into specific studies dedicated to GC resistance revealed the following responsible mechanisms:

- Deficient binding between the GC and the GR or between the GR complex and DNA may be a cause [39].
- Increased antagonism is determined either by increased GR-β expression [40] or by diminished GR-α expression [41]. This can be explained by the IL-2/IL-4-induced suppression of GR-α (and not GR-β) expression in peripheral blood mononuclear cells (PBMCs) [42]. Additionally, IL-2 and IL-4 can synergistically reduce (via the p38MAPK pathway) nuclear translocation and binding affinity in T-cells (reversible by a p38 inhibitor) [43]. Furthermore, IL-17 and IL-23 cytokines were reported to significantly upregulate GR-β [42].
- Inflammation or oxidative stress has the potential to negatively affect GC signaling [22].
- The expression of various anti-inflammatory genes induced by GCs can be reduced through GR phosphorylation by, for example, p38 mitogen-activated protein kinase (MAPK) and by the reduced activity of histone deacetylase 2 (HDAC2) [43,44].
- The upregulation of certain cytokines, such as IL-2, IL-4, and IL-13, was detected in the lungs of patients with GC unresponsiveness [45,46,47]; in vitro, the overexpression of these cytokines was associated with the phosphorylation of GR and a decrease in nuclear translocation in inflammatory cells through the activation of p38 mitogen-activated protein kinase [48]. p38MAPK activity was demonstrated to be higher in alveolar macrophages from patients with impaired response to GCs compared to ‘responders’. Furthermore, the expression of MKP-1 (DUSP1 gene), an endogenous inhibitor of the MAPK pathway, was significantly diminished in alveolar macrophages after GCS exposure, leading to an increase in p38MAPK activity [49]. Furthermore, p38MAPK inhibitors, such as AZD7624 or SB203580, have recently been investigated in corticosteroid-resistant asthmatic populations [50,51].
- Increased HDAC activity using theophylline, PI3K, and p38 MAPK inhibitors demonstrated beneficial effects [52,53,54], especially in glucocorticoid-resistant asthmatic smokers, where increased antagonism of the GR-α resulted from a reduced ratio of GR-α to GR-β isoforms [55]. Moreover, reduced total HDAC activity in PBMCs isolated from prednisone-dependent asthmatics compared to ICS-maintained moderate asthmatics and healthy volunteers was reported [56].
- GC resistance has been associated with *Haemophilus influenzae*, *Chlamydia pneumoniae*, *Influenza A virus* (IAV), rhinovirus, and *Respiratory syncytial virus* (RSV) infections [57,58,59,60,61]. The molecular mechanism proposed for glucocorticoid insensitivity in rhinovirus-infected primary human bronchial epithelial cells is the activation of NF-κB and c-Jun N-terminal kinase, which leads to a decrease in GR-α nuclear translocation [62]. The influence of NF-κB activity on GC resistance has also been confirmed by research on the blockade of this pathway [63,64].
- Using mouse models of steroid-resistant asthma driven by bacterial (*Chlamydia* and *Haemophilus influenzae*) and viral (*influenza* and RSV) respiratory tract infections, Kim et al. demonstrated that steroid insensitivity can be induced through PI3K-mediated phosphorylation and the nuclear translocation of pAKT [65].
- By upregulating miR-9 expression in pulmonary macrophages, IFN-γ can increase GR phosphorylation and, consequently, inhibit GR nuclear translocation in experimental models of steroid-resistant airway hyperresponsiveness [66].
- In a study of human fetal airway smooth muscle cells, TNF-α and IFN-γ cytokines were shown to sustain GC resistance by promoting the Nuclear factor-κB (NF-κB) pathway and Stat1 phosphorylation [67]. TNF-α also demonstrated the potential to activate the c-Jun N-terminal kinase (JNK), which directly phosphorylated GR-α at Ser226 and inhibited GRE-binding [68].
- The nitrosylation of the glucocorticoid receptor at the HSP90 (chaperone) binding site can be caused by high levels of nitric oxide generated in situ as a result of eosinophilic inflammation. This can decrease its affinity with chaperone proteins that protect it from cytoplasmic degradation. The binding affinity to GCs (ligand) in structural cells, such as fibroblasts, can also be reduced by nitrosylation [69]. In conclusion, asthmatics with persistent airway eosinophilia with increased localized nitric oxide production and possibly increased remodeling may develop GC resistance through the repeated nitrosylation of GR.
- Increased NLR Family Pyrin Domain Containing 3 (NLRP3) inflammasome/IL-1β activation contributed to glucocorticoid resistance in murine models of steroid-resistant allergic airway disease [70].
- The Th2 cytokines IL-13 and IL-5 each possess the ability to induce diminished GR-binding affinity. The effect of hydrocortisone in suppressing LPS-induced IL-6 production by monocytes was demonstrated to be significantly hindered when the cells were primed by IL-13 [71]. Additionally, IL-5-primed eosinophils were unresponsive to GS-induced apoptosis (via synergistic upregulation of nuclear-factor IL-3 due to a cross-talk between GCS-induced trans-activation signaling and IL-5 antiapoptotic pathway) [72].
- The adoptive transfer of Th17 cells in mice resulted in the development of steroid insensitivity, and Th17 cells and IL-17A levels are frequently associated with CG resistance in asthmatic patients [73,74,75]. Accordingly, the expression of GR-β has been reported to increase Th17 responses [76]. In the obesity phenotype of asthma, the associated steroid resistance may be induced by IL-17 produced by the pulmonary type 3 innate lymphoid cells [77]. The role of IL-7 in GC resistance has been confirmed by the augmentation of dexamethasone anti-inflammatory action in diesel exhaust particle-induced neutrophilic steroid insensitivity secondary to anti-IL-17 therapy [78].
- Bhavsar et al. showed that dexamethasone could not suppress the lipopolysaccharide (LPS)-induced release of pro-inflammatory cytokines [49]. This finding was supported by Li et al., who simulated an airway infection in a mouse model of steroid-resistant asthma through the concomitant administration of LPS + IFNγ; consequently, PP2A activity (that induced JNK) was attenuated and led to the phosphorylation of GR-α at Ser226, thereby hindering glucocorticoid receptor nuclear translocation in pulmonary macrophages [66].
- LPS promoted a shift from Th2-derived airway eosinophilic inflammation to Th17-drived neutrophilic inflammation in an ovalbumin-sensitized murine asthma model [79].
- Dysregulated IL-10 production is associated with GC insensitivity. This is probably due to impaired IL-10 production, according to Hawrylowicz et al., who compared in vitro stimulated T lymphocytes from corticosteroid-resistant asthmatic with dexamethasone to T lymphocytes from steroid-sensitive asthmatics [80].
- The induction of Th2/Th17 responses in fungus-exposed patients has the potential to develop GC resistance [65]. More precisely, in neonatal mice, *Aspergillus alternata* exposure induced IL-33 dependent GC resistant asthma, mediated by ILC2 and Th2 cells [81]. The suggested mechanism underlying glucocorticoid insensitivity is the activation of p38-MAPK in CD4 + T cells and induction of phosphorylation of GR by IL-33 [82].

GCs also produce pro-inflammatory effects under stress conditions [83]. Table 2 depicts the potential targeted molecular and immunopathogenic mechanisms in glucocorticoid-resistant severe asthma.

## 4. Potentially Therapeutic Effect of Marine-Derived Biologically Active Compounds in Severe Asthma 

Experimental studies with marine compounds demonstrating their effectiveness in in vitro or in vivo models of bronchial asthma are scarce [84,85,86,87,88]. On the other hand, recent studies revealed the significant potential of marine compounds to interfere with molecular mechanisms similar to those involved in GC-resistant asthma. Therefore, a standardized inclusion–exclusion criterion was implemented, aiming to justify the current review. The literature search queries were performed until September, 2021. We included and analyzed all the original articles from PubMed and Scopus databases, with marine compounds that potentially target these molecular mechanisms; we excluded reviews and generalized or irrelevant studies (results illustrated in Table 3).

Some of these compounds from different marine sources are well characterized and have well-defined structures (Table 4), while others are extracts with complex compositions.

### 4.1. Cellular Signal/Corticoresistance

Fucosterol, a phytosterol from the marine brown algae *Padina boryana*, demonstrated anti-inflammatory effects through its dose-dependent downregulation of pro-inflammatory cytokines (IL-1β, IL-6 and TNF-α) and of the Nrf2/HO-1 pathway [89].

Mojabanchromanol (MC), a chromanol isolated from the brown algae *Sargassum horneri*, demonstrated anti-oxidant effects through the attenuation of particulate matter-induced oxidative stress, the reduction of the ROS-mediated phosphorylation of MAPK extracellular signal-regulated kinase 1/2 (Erk1/2) and of c-JNK, and the inhibition of the secretion of pro-inflammatory cytokines (IL-6, IL-1β and IL-33). The authors proposed that mojabanchromanol be developed as a therapeutic agent against particulate matter-induced airway inflammatory responses [84].

Sargachromenol, isolated from *Sargassum horneri*, demonstrated anti-inflammatory effects in lipopolysaccharide (LPS)-stimulated RAW 264.7 macrophages, by reducing the nitric oxide (NO); and in intracellular reactive oxygen species (ROS), by decreasing the mRNA expression levels of inflammatory cytokines (IL-1β, IL-6, and TNF-α) and by inhibiting the activation of NFκB and MAPK signaling [90].

Fucoidan, purified from *Saccharina japonica*, reduced the production of NO, and downregulated the expression of the MAPK (including p38, ENK and JNK) and NF-κB (including p65 and IKKα/IKKβ) signaling pathways in a zebrafish experiment [91].

Using free-radical-scavenging assays antioxidant properties were discovered for the sesquiterpenoids in green algae *Ulva fasciata* Deliles [92,93].

An extract from Korean marine alga *Ulva pertusa*, 4-hydroxy-2,3-dimethyl-2-nonen-4-olide, moderately inhibited the release of the pro-inflammatory cytokines IL-12 p40 and IL-6 from bone marrow-derived dendritic cells, as well as signal transduction by inhibiting phosphorylation of NF-kB, and, thus, warranted further study to evaluate its potential as a “therapeutic agent for inflammation-associated maladies” [94].

Two fatty acids, (E)-9-Oxooctadec-10-enoic-acid and (E)-10-Oxooctadec-8-enoic-acid, isolated from *Gracilaria verrucosa*, inhibited the production of inflammatory biomarkers, including NO, IL-6, and TNF-α, by suppressing the nuclear translocation of NF-kB and the phosphorylation of STAT1 in LPS-stimulated RAW264.7 cells [95].

Three diketopiperazine derivatives, cyclo(L-Pro-D-Val), cyclo(L-Pro-L-Tyr), and cyclo(L-pro-D-Leu), derived from two marine bacteria, *Bacillus* sp. *HC001* and *Piscicoccus* sp. *12L081*, demonstrated anti-inflammatory effects through the inhibition of p38 MAPK activation and the downregulation of TNF-α, IL-6, NF-kB, and ERK1/2 [96].

Cycloprodigiosin, an analog of prodigiosin obtained from *Pseudoalteromonas dentrificans,* inhibited TNF- α induced NF-kB activation, in HeLa, U373, and COS7 cell lines [97,98].

C-Phycocyanin from *Nostoc Muscorum* cyanobacteria is a pigment with antioxidant potential [99,100].

Pyrenocine A, produced from the marine-derived fungus *Penicillium paxilli*, produces immunosuppressive effects through the inhibition of pro-inflammatory mediators (TNF-α and PGE2) and inhibits the expression of genes related to NFκB activation in macrophages stimulated with LPS [108].

Two new components, (−)-1S-myrothecol and (+)-1R-myrothecol, isolated from the deep-sea fungus *Myrothecium* sp. *BZO-L062*, presented anti-inflammatory and antioxidant activities [109].

Oscarellin, an anthranilic acid derivative isolated from *Oscarella stillans*, a Philippine sponge, strongly inhibits LPS-induced TNF-α and IL-6 production in murine macrophage RAW264.7. These changes are associated with the inactivation of c-Jun NH2-terminal kinase (JNK), extracellular signal-regulated kinase (ERK), activator protein-1 (AP-1), and NF-kB, and the activation of activating transcription factor-3 (ATF-3) [115].

Lobocrassin B, an extract from the soft coral *Lobophytum crissum*, inhibited the production of TNF-α and NF-κB, an important transcription factor responsible for cytokine production, in mouse dendritic cells [116].

Didemnin B (Depsipeptides) was extracted and isolated from *Trididemnum solidum*; it exhibited strong anti-inflammatory and immunosuppressive activity through the inhibition of iNOS and NF-kB [122,123].

*Sargassum horneri* ethanol extract, from an edible brown marine algae, demonstrated anti-inflammatory effects mediated by the phosphorylation of MAPK p38, extracellular signal-regulated kinase 1/2 (Erk1/2), and c-JNK, to induce the particulate matter-induced mRNA expression of pro-inflammatory cytokines (IL-1β, TNF-α, IL-6), lung epithelial cell derived-chemokines (IL-8, MCP-1, and chemokine (CCL5), and to suppress the mRNA expression of particulate matter-induced pro-allergic cytokines thymic stromal lymphopoietin (TSLP) and IL-33 [126].

*Sargassum horneri (Turner) C. Agardh* ethanol extract (SHE), obtained from the brown algae *Sargassum horneri*, demonstrated anti-inflammatory and cytoprotective effects on macrophage cells as a model for alveolar lung cells, probably via the p38 MAPK pathway and Nrf2/HO-1 expression. The extract inhibited the production of inflammatory mediators (iNOS, COX-2, and PGE2) and pro-inflammatory cytokines (IL-1β, IL-6, and TNF-α) [127]. 

Another ethanol extract with a commercial grade of 70%, separated from *Sargassum horneri*, demonstrated similar effects: it significantly repressed the secretions of inflammatory cytokines and reduced protein expression in PGE2, TNF-α, IL-6, IL-1β, NF-κB, and MAPKs from PM-activated macrophages [128].

A sulfated polysaccharide with a sulfate content of 9.07% from *Saccharina japonica* showed significant inhibition of NO and PGE2 production via the downregulation of iNOS and COX-2 expression. The polysaccharide also suppressed TNF-α and (IL)-1β production via the NF-κB and MAPK signal pathways in LPS-induced RAW cells [131].

A sulfated polysaccharide isolated from *Sargassum fulvellum* demonstrated a significant and concentration-dependent decrease in the production levels of NO, TNF-α, PGE2, IL-6, and IL-1β in LPS-treated RAW 264.7 macrophages [132].

The fatty acids from the *Arctoscopus japonicus* egg of a cold-water marine fish presented anti-inflammatory effects through the suppression of the expression of iNOS, COX-2, IL-1β, IL-6, and TNF-α, and a reduction in the phosphorylation levels of NF-κB p-65, p38, ERK1/2, and JNK, key components of the NF-κB and MAPK pathways [139].

### 4.2. Cytokine Levels

#### 4.2.1. Th2 Cytokines

The whole culture extract of a marine-derived actinomycete strain, culture CNQ431, identified as a *Streptomyces* sp., demonstrated potent suppression of Th2 cytokines IL-5 and IL-13, but also the production of the dendritic cell-associated cytokines IL-1 and TNF-α, indicating immunosuppressive effects on both the APCs (i.e., dendritic cells) and the Th2 cells in a mouse splenocyte assay [124].

An ethanol extract from *Sargassum horneri*, obtained from a brown alga, was found to have antioxidant, anti-inflammatory, and anti-allergic effects in a BALB/c mouse model of asthma sensitized with ovalbumin. IL-4, IL-5, and IL-13 were found to be decreased in the lungs of PM-exacerbated asthmatic mice. Concomitantly, the Th17 cell response, the expression of responses of relevant effector cytokines, IL-17a and Th2/Th17, were also decreased [86].

A methanol extract of *Sargassum hemiphyllum*, a brown seaweed, inhibited the increase of TNF-α-induced NF-kB protein levels, the transcription factor of TNF-α, and IL-8 and TNF-α release, suggesting an inhibitory effect on atopic allergic reactions [130].

Exopolysaccharide EPCP1-2, an extracellular polysaccharide extracted from *Crypthecodinium cohnii,* has significant potential to inhibit macrophage proliferation, as well as to downregulate the expression of TLR4, TAK1, MAPKs, and NF-κB protein. It acts as an anti-inflammatory agent through macrophage suppression on the RAW 264.7 macrophage cell line and is a potent regulatory MAPK, and NF-κB signaling pathways [133].

Spirulina extract (Immulina^®^), a high-molecular-weight polysaccharide extract from the cyanobacterium *Arthrospira platensis* (Spirulina), showed anti-inflammatory and inhibitory effects in an induced allergic inflammatory response and on histamine release from RBL-2H3 mast cells. It also has the potential to inhibit the IgE-antigen-complex-induced production of TNF-α, IL-4, leukotrienes, and histamine, and showed promising effects with respect to the relief of allergic rhinitis symptoms [137,138].

A component of this extract, n-hexane, a fatty-acid-rich fraction, ameliorated allergic airway inflammation in a mouse model of ovalbumin-induced asthma: eosinophil infiltration and goblet cell hyperplasia were significantly reduced around the airways, and the concentrations of Th2-related cytokines (IL-4, IL-5, and IL-13) and Th17-related cytokines (IL-17) were significantly decreased in the spleen and bronchoalveolar lavage fluid [88].

Phlorotannins isolated from brown algae, *Eckolonia cava*, exhibited anti-allergic activities through the inhibition of degranulation: the tested compounds suppressed the binding between IgE and FcεRI receptors [140].

Reticulol, a polyketide isolated from *Graphostroma* sp. MCCC 3A00421 deep-sea hydrothermal sulfide deposits from the Atlantic Ocean, showed potent inhibition of degranulation with an IC50 value of 13.5 µM [141].

Three compounds isolated from the deep-sea-derived marine *Williamsia* sp. MCCC 1A11233 (CDMW), CDMW-3, CDMW-5, and CDMW-15, demonstrated antiallergic activity due to the block of mast-cell-dependent passive cutaneous anaphylaxis in IgE-sensitized mice and to the decrease of degranulation and histamine release in immunoglobulin E (IgE)-mediated rat basophilic leukemia (RBL)-2H3 cells [142].

An extract of *Apostichopus japonicus*, obtained from a sea cucumber, showed anti-oxidant and anti-inflammation effects in mice with ovalbumin-induced asthma. The hyper-responsiveness of airways was significantly lower, the number of eosinophils in the lungs was decreased, and T regulatory cells significantly increased in the mesenteric lymph nodes [143].

Peridinin and fucoxanthin, carotenoids isolated from *Symbiodinium* sp., a symbiotic dinoflagellate, and from *Petalonia fascia*, a brown alga, respectively, were shown to suppress allergic inflammatory responses through the inhibition of delayed-type hypersensitivity in mice, and to reduce the number of eosinophils in both the ear lobe and peripheral blood. The inhibitory effect of peridinin was higher than that of fucoxanthin [144].

#### 4.2.2. Th17/Non Th2 Cytokines

Two novel phenazines, obtained from marine-derived *Streptomyces* sp., showed anti-inflammatory potential and inhibited the production of LPS-induced NO, TNF-α-induced NFkB activity [101,102,103,104].

Griseusrazin A, a pyrazine derivative produced from marine *Streptomyces griseus*, inhibited the production of pro-inflammatory cytokines, such as IL-1β, IL-6, and TNF-α, in LPS-stimulated cells and suppressed iNOS [103,105].

Grassystatin A, obtained from the cyanobacterium *Lyngbya confervoides*, inhibited the upregulation of IL-17 and interferon-γ (INF-γ) in response to antigen presentation, reduced T cell proliferation in a dose-dependent manner, and inhibited the upregulation of IL-17 and IFN-γ in response to antigen presentation [106].

Ogipeptins A-D, obtained from the culture broth of the Japanese marine Gram-negative bacterium *Pseudoalteromonas* sp. SANK 71903, decreased TNF-α release by human U937 monocytic cells [107].

Three chrysamides, A–C, dimeric nitrophenyl trans-epoxyamides obtained from the deep-sea-derived fungus, *Penicillium chrysogenum* SCSIO41001, suppressed the production of proinflammatory cytokine IL-17 [110].

Brevicompanine E, isolated from a deep ocean sediment-derived fungus, *Penicillium* sp., was found to inhibit LPS-induced TNF-α, IL-1β, iNOS and COX-2 production in microglia and LPS-induced IκBα degradation, NF-κB nuclear translocation, and Akt, c-Jun NH2-terminal kinase (JNK) phosphorylation [111].

Bolinaquinone, a polyoxygenated sterol derived from *Dysidea* sp., inhibited neutrophilic infiltration and IL-1β and PGE2 levels [112,113].

Hirsutanol A (HA), isolated from a red alga-derived fungus, *Chondrostereum* sp. NTOU4196, significantly attenuated the levels of TNF-α, IL-6, and IL-1β in LPS-treated THP-1 cells [114].

Carijoside, a steroid glycoside extracted from *Carijoa sp*., inhibited the superoxide anion generation and elastase release by human neutrophils [117]. On the other hand, rossinones A and B, terpene-derived metabolites of the *Aplidium speciesascidian* family of *Polyclinidae*, inhibited only the superoxide production [120].

Klyflaccisteroid J, a steroid isolated from the Formosan soft coral *Klyxum flaccidum*, demonstrated suppression of N-formyl-methionyl-leucylphenyl-alanine/cytochalasin B (fMLP/CB)-induced superoxide anion generation and elastase release in human neutrophils in vitro [118]. New steroids, klyflaccisteroids K–M, also isolated from *Klyxum flaccidum*, demonstrated the suppression of superoxide anion generation and elastase release [119].

A brominated indol isolated form the gastropod mollusk *Dicathais orbita*, 6-bromoisatin, inhibited inflammation in a murine model of LPS-induced acute lung injury by significantly reducing TNF-α and IL-1β production and associated lung damage [121].

Sinulerectol A and Sinulerectol B, as extracts isolated from the marine soft coral *Sinularia erecta*, showed anti-inflammatory activities in neutrophil pro-inflammatory responses [125]. 

*Sargassum horneri* (Turner), a C. Agardh ethanolic extract with 70% ethanol lyophilized at −40 °C, obtained from a brown alga, showed antioxidant and anti-inflammatory effects through a dose-dependent reduction of the mRNA level of cytokines, including IL-1β, and pro-inflammatory genes, such as iNOS and COX-2, in LPS-stimulated macrophage activation. In addition, the anti-inflammatory effects were obtained by inhibiting ERK, p-p38 and NF-κB phosphorylation and by the release of IL-1β in LPS-stimulated macrophages [129].

A cyanobacterial lipopolysaccharide, isolated from *Oscillatoria planktothrix* FP1, demonstrated anti-inflammatory effects through the inhibition of LPS-induced IL-1β, TNF-α, and IL-8 production [134,135,136].

## 5. Conclusions

In recent years, efforts have been made to understand the mechanisms underlying GC resistance. To overcome GC resistance, which is frequently associated with high doses of GC treatment, there is an urgent need for more specific targeted therapies. Natural compounds have been demonstrated to be effective against various pathological mechanisms through a variety of pathways. Some of these mechanisms were also shown to be involved in GC resistance. This paper reviewed the marine compounds potentially acting on the mechanisms involved in GC-resistant severe asthma. The article provided a basis for the development of effective marine-derived drugs as new and safe sources for the potential treatment of glucocorticoid-resistant severe asthma. 

## Figures and Tables

**Table 1:** Degree of Glucocorticoid Resistance and Corresponding Asthma Phenotypes.

Degree of Glucocorticoid Resistance	Asthma Phenotypes	Pathobiologic Features
Severe corticosteroid resistance	Obesity-related asthma	Absence of Th2 specific response Increased oxidative stress
	Neutrophilic asthma	Increased Th-17 response (increased IL-8, neutrophilia)
	Late-onset eosinophilic asthma	Increased IL-5 Eosinophilia
Moderate corticosteroid resistance	Early-onset allergic asthma	Increased Th2 specific response Presence of antigen-specific IgE
	Exercise-induced asthma	Increased Th2 specific response Increased mast cells degranulation Increased CysLTs

Th, T helper lymphocyte; Ig, Immunoglobuline; IL, interleukin; CysLTs, cystenyl leukotrienes.

**Table 2:** Potential targeted mechanisms in glucocorticoid-resistant severe asthma.

Molecular Targets	Pharmacological Effect	References
Decrease in activity of MAPK	Decrease in GR phosphorylation Increased ratio of GR-α to GR-β isoforms	[43,44,48,49,50,51,52,53,54,55]
Increase of activity of HDAC	Decrease in GR phosphorylation Increased ratio of GR-α to GR-β isoforms	[43,44,52,53,54,56]
Decrease in activation of JNK	Decrease in GR phosphorylation Increase in GR-α nuclear translocation	[68] [62]
Nitric oxide decrease	Decrease in nitrosylation of GR at HSP90 (chaperone binding site)	[69,84]
Decrease inactivation of NF-κB	Increase in GR-α nuclear translocation	[62,63,64,67]
Decrease in oxidative stress	Multiple	[22]
Downregulation of Th2		
IL-4	Increase in GR-α expression and nuclear translocation Increase in GR binding affinity in T-cells Decrease in GR phosphorylation	[40,41,42,43,45,46,47,48]
IL-5	Increased GR binding affinity	[71,72]
IL-13	Decrease in GR phosphorylation Increased GR binding affinity	[45,46,47,48] [71]
Downregulation of non-Th2		
IL-17	Decrease in GR-β expression	[42,76,77]
IL-23	Decrease in GR-β expression	[42]
IFN-γ	Decreased GR phosphorylation and stimulation of GR nuclear translocation Increase in GR-α nuclear translocation (through downregulation of NF-κB)	[66] [67]
TNF-α	Increase in GR-α nuclear translocation (through downregulation of NF-κB) Decrease in GR-α phosphorylation at Ser226 and the inhibition of GRE-binding (through downregulation of JNK)	[67] [68]
IL-33	Decreased GR phosphorylation	[81,82]
IL-1β	Unknown	[70]
Inhibition of inflammatory response shift: Th2 to Th17 ^†^	Decreased GR phosphorylation and stimulate GR nuclear translocation Increase in GR-α nuclear translocation (through downregulation of NF-κB) Decrease in GR-α phosphorylation at Ser226 and the inhibition of GRE-binding (through downregulation of JNK)	[49,66]

MAPK, Mitogen-activated protein kinase; GR, glucocorticoid receptor; HDAC, histone deacetylase; JNK, c-Jun N-terminal kinase; HSP90, heat shock protein; NF-κB, Nuclear factor-κB; ^†^, inhibition of inflammatory response shift from Th2-derived airway eosinophilic inflammation to Th17-drived neutrophilic inflammation (through inhibition of LPS-induced release of pro-inflammatory cytokines).

**Table 3:** Potential targeted mechanisms in glucocorticoid-resistant severe asthma.

Compound	Specie	Origin	Class	Molecular Targets																References
				Decrease Activity of MAPK	Increase of Activity of HDAC	Decrease Activation of JNK	Nitric Oxide Decrease	Decrease Activation of NF-κB	Decrease Oxidative Stress	Downregulation of Th2			Downregulation of Non-Th2							
										IL-4	IL-5	IL-13	IL-17	IL-23	IFN-γ	TNF-α	IL-33	IL-1β	Inhibition of Inflammatory Response Shift: Th2 to Th17 ^†^	
Simple compounds																				
Fucosterol	*Padina boryana*	brown algae	phytosterol	x			x	x								x		x		[89]
Mojabanchromanol	*Sargassum horneri*	brown algae	chromanol	x		x			x								x	x		[84]
Sargachromenol	*Sargassum horneri*	brown algae	chromenol	x			x	x												[90]
Fucoidan	*Saccharina japonica*	brown algae	polysaccharides	x		x	x	x												[91]
3,4,5,5-Tetramethyl-4-(3′-oxopentyl)-2-cyclohexen-1-one	*Ulva fasciata* Deliles	green algae	sesquiterpenoids						x											[92,93]
4-hydroxy-2,3-dimethyl-2-nonen-4-olide	*Ulva pertusa*	green algae	extract					x												[94]
(E)-9-Oxooctadec-10-enoic-acid and (E)-10-Oxooctadec-8-enoic-acid	*Gracilaria verrucosa*	red algae	fatty acids				x	x								x				[95]
cyclo(L-Pro-D-Val), cyclo(L-Pro-L-Tyr), cyclo(L-pro-D-Leu)	*Bacillus* sp. *HC001*, *Piscicoccus* sp. *12L081*	bacteria	diketopiperazine	x				x								x				[96]
Cycloprodigiosin	*Pseudoalteromonas dentrificans*	bacteria	prodigiosin					x								x				[97,98]
C-Phycocyanin	*Nostoc Muscorum Cyanobacteria*	bacteria	Polypeptide						x											[99,100]
Phenazines 1,2	*Streptomyces* sp.	bacteria	aromatic secondary metabolites				x	x								x				[101,102,103,104]
Griseusrazin A	*Streptomyces griseus*	bacteria	pyrazine-type molecules				x	x								x		x		[103,105]
G rassystatin A	*Lyngbya confervoides*	bacteria	linear decadepsipeptide										x							[106]
Ogipeptins A-D	*Pseudoalteromonas* sp. *SANK 71903*	bacteria	cyclic peptides													x			x	[107]
pyrenocine A	*Penicillium paxilli*	fungus	phytotoxins					x								x				[108]
(−)-1S-myrothecol and (+)-1R-myrothecol	*Myrothecium* sp. *BZO-L062*	fungus	alkylresorcinol						x											[109]
Chrysamides A–C	*Penicillium chrysogenum SCSIO41001*	fungus	dimeric nitrophenyl trans-epoxyamides												x					[110]
Brevicompanine E	*Penicillium* sp.	fungus	diketopiperazine derivatives			x	x	x								x		x	x	[111]
Polyoxygenated Sterols & bolinaquinone	*Dysidea* sp.	fungus	sterols															x		[112,113]
Hirsutanol A	*Chondrostereum* sp. *NTOU4196*	fungus	Sesquiterpene compound													x		x	x	[114]
Oscarellin	*Oscarella stillans*	sponge	anthralinic acids			x		x								x				[115]
Lobocrassin B	*Lobophytum crassum*	coral	diterpenoids					x								x				[116]
Carijoside A	*Carijoa* sp.	soft coral	Steroid glycoside						x											[117]
Klyflaccisteroid J	*Klyxum flaccidum*	soft coral	steroidal derivatives						x											[118]
Klyflaccisteroid K-M	*Klyxum flaccidum*	soft coral	steroidal derivatives						x											[119]
Rossinones A & B	*Aplidium speciesascidian*	marine animals	Terpene derived metabolite						x											[120]
6-bromoisatin	*Dicathais orbita*	gastropod mollusc	brominated indole derivatives													x		x		[121]
Didemnin B	*Trididemnum solidum*	ascidia	depsipeptides				x	x												[122,123]
Splenocins A-I	*Streptomyces* sp.	bacteria	depsipeptides								x	x				x		x		[124]
Sinulerectol A & B	*Sinularia erecta*	soft coral	cembranoid						x											[125]
Complex composition																				
*Sargassum horneri* extract	*Sargassum horneri*	brown algae	extract	x		x										x	x	x		[126]
*Sargassum horneri (Turner)* ethanol extract	*Sargassum horneri (Turner) C. Agardh*	brown algae	extract	x			x		x							x		x		[127]
*Sargassum horneri (Turner)* ethanol extract	*Sargassum horneri*	brown algae	extract	x				x								x		x		[128]
*Sargassum horneri* ethanol extract	*Sargassum horneri*	brown alga	ethanol extract						x	x	x	x	x							[86]
*Sargassum horneri (Turner) C. Agardh* ethanol extract	*Sargassum horneri (Turner) C. Agardh*	brown algae	ethanolic extract	x			x	x										x	x	[129]
*Sargassum hemiphyllum* methanol extract	*Sargassum hemiphyllum*	brown alga	methanol extract					x								x				[130]
Sulfated polysaccharide	*Saccharina japonica*	brown algae	polysaccharides	x			x	x	x							x		x		[131]
Sulfated polysaccharide	*Sargassum fulvellum*	brown algae	polysaccharides				x		x							x		x		[132]
Exopolysaccharide (EPCP1-2)	*Crypthecodinium cohnii*	microalgae	polysaccharide	x				x												[133]
Cyanobacterial lipopolysaccharide	*Oscillatoria planktothrix FP1*	bacteria	lipopolysaccharides													x		x		[134,135,136]
Spirulina extract	*Arthrospira platensis (Spirulina)*	bacteria	lipoproteins							x						x				[137,138]
*Arctoscopus japonicus* egg extracted lipids	*Arctoscopus japonicus*	fish	fatty acid	x		x	x	x								x		x		[139]
*Apostichopus japonicus* extract	*Apostichopus japonicus*	marine animals	extract							x	x	x	x							[88]

^†^, inhibition of inflammatory response shift from Th2-derived airway eosinophilic inflammation to Th17-drived neutrophilic inflammation (through inhibition of LPS-induced release of pro-inflammatory cytokines).

**Table 4:** Chemical formula of marine drugs with potential use in glucocorticoid-resistant severe asthma.

Compound	Chemical Structure
fucosterol	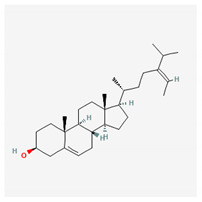
mojabanchromanol	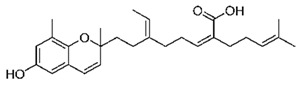
sargachromenol	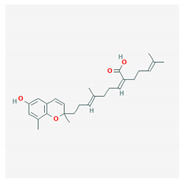
fucoidan	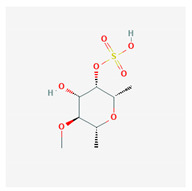
3,4,5,5-Tetramethyl-4-(3′-oxopentyl)-2-cyclohexen-1-one	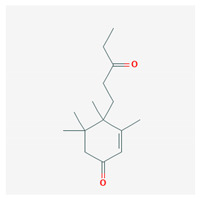
4-hydroxy-2,3-dimethyl-2-nonen-4-olide	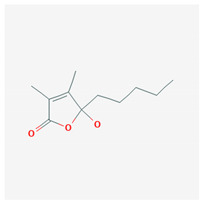
(E)-9-Oxooctadec-10-enoic-acid and (E)-10-Oxooctadec-8-enoic-acid	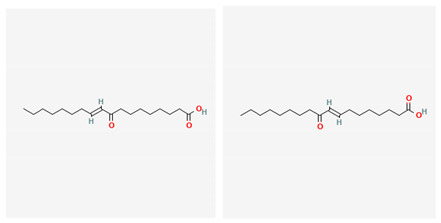
cyclo(L-Pro-D-Val), cyclo(L-Pro-L-Tyr), cyclo(L-pro-D-Leu)	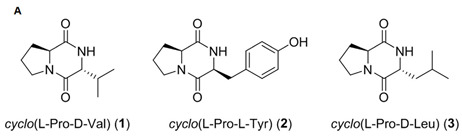
cycloprodigiosin	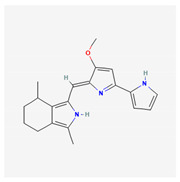
C-phycocyanin	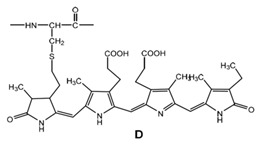
phenazines 1,2	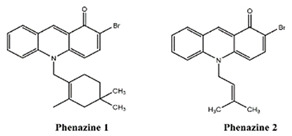
griseusrazin A	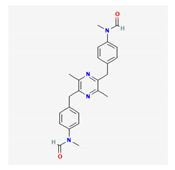
grassystatin A	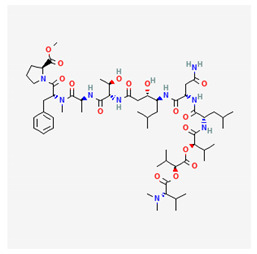
ogipeptins A-D	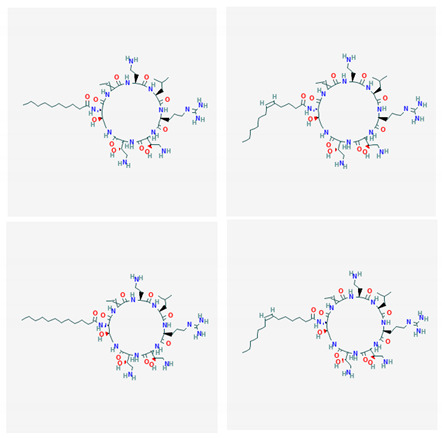
pyrenocine A	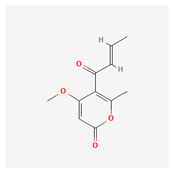
(−)-1S-myrothecol and (+)-1R-myrothecol	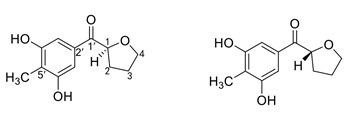
chrysamides A–C	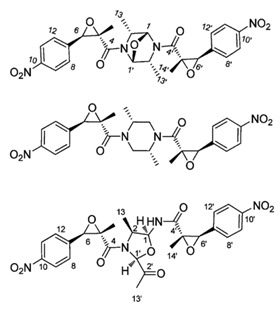
brevicompanine E	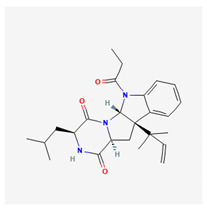
polyoxygenated dysidea sterols	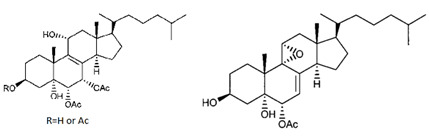
bolinaquinone	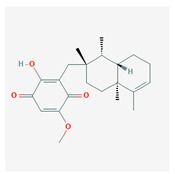
hirsutanol A	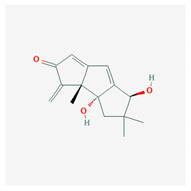
oscarellin	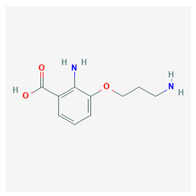
lobocrassin B	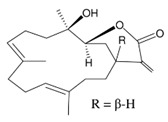
carijoside A	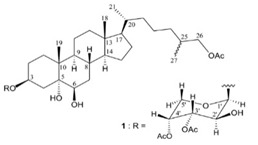
klyflaccisteroid J	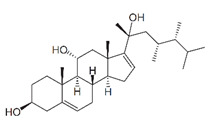
klyflaccisteroid K-M	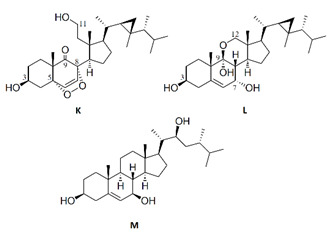
rossinones A & B	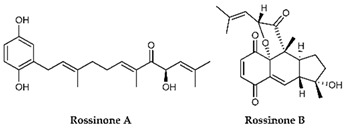
6-bromoisatin	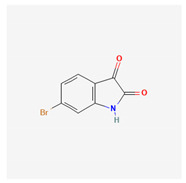
didemnin B	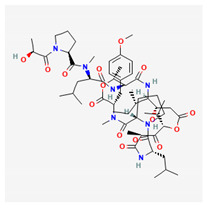
splenocins A-I	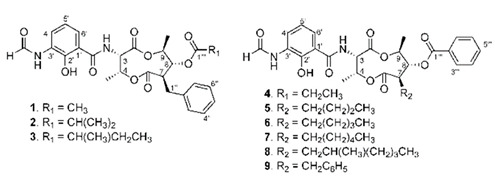
sinulerectol A & B	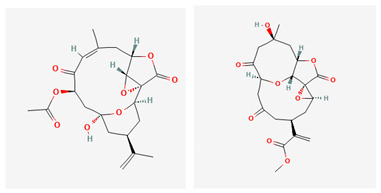
